# Predictive Factors for Smoking Cessation Among People With Lung Cancer Attending French Cessation Services, According to Sex

**DOI:** 10.1016/j.jtocrr.2025.100888

**Published:** 2025-08-07

**Authors:** Anne-Laurence Le Faou, Dalia Alleaume, Ingrid Allagbé

**Affiliations:** a Outpatient Addictology Center, Georges Pompidou European Hospital, Assistance Publique – Hôpitaux de Paris (AP-HP), Université Paris Cité, Paris, France; b Faculty of Medicine, Université Paris Cité, Paris, France; c Association Robert Debré pour la Recherche Médicale, Paris, France

**Keywords:** Sex, Smoking cessation, Lung cancer, France, Smoking cessation services

## Abstract

**Background:**

Limited research exists on sex-specific smoking cessation interventions for patients with lung cancer. This study leverages data from the Consultations de Dépendance Tabagique, the French national database of smoking cessation services (SCS), to identify sex-specific factors influencing smoking cessation in people with lung cancer.

**Methods:**

This retrospective observational study analyzed data from 3407 adults with lung cancer (31.2% women, 68.8% men) registered in the Consultations de Dépendance Tabagique between 2001 and 2018. Participants were people with active tobacco use with at least one follow-up SCS consultation. The primary outcome was 28-day smoking abstinence, confirmed by exhaled carbon monoxide less than 10 parts per million. Multivariate logistic regression identified predictors of abstinence, stratified by sex.

**Results:**

Abstinence rates were similar in women (35.2%) and men (35.4%) (*p* = 0.40). Women had higher psychological distress (19.8% with depression versus 13.1% in men; *p* < 0.001) and were more likely to seek SCS independently (19.4% versus 13.6%; *p* < 0.001). Men smoked more cigarettes daily (27 versus 25; *p* = 0.002) and had higher alcohol consumption (35.7% versus 13.9%; *p* < 0.001). Confidence in quitting (women: odds ratio [OR] = 1.91; 95% confidence interval [CI]: 1.27–2.87; men: OR = 1.50; 95% CI: 1.16–1.95) and follow-up consultations (≥7: women: OR = 8.86; 95% CI: 5.69–14.0; men: OR = 6.64; 95% CI: 4.88–9.13) predicted abstinence for both sexes. Among women, hospital referral (OR = 1.63; 95% CI: 1.10–2.43) and living with other persons who smoke (OR = 4.16; 95% CI: 1.70–10.4) increased abstinence, whereas in men, nicotine replacement therapy (OR = 1.46; 95% CI: 1.09–1.97) was beneficial.

**Conclusions:**

The results indicate a need for further research into targeted interventions by sex to evaluate the efficacy of smoking cessation strategies in patients with lung cancer.

## Introduction

Sex-specific factors can influence smoking cessation outcomes in individuals with lung cancer, driven by biologic, psychologic, and social differences.^1^, ^2^, ^3^ Historically, women have received less attention in smoking cessation disparity research, possibly because men have traditionally had higher smoking rates worldwide.^4^^,^^5^ However, recent trends indicate a decline in smoking prevalence among men, whereas smoking among women is increasing. This has led to an increase in lung cancer diagnoses among women in high-income countries.^6^

Physiologically, differences in nicotine metabolism and receptor sensitivity can impact dependency levels and withdrawal experiences between sexes.^1^ Notably, women have considerably higher rates of nicotine clearance than men, contributing to sex-based differences in responses to smoking cessation medications.^7^^,^^8^ Furthermore, women often experience higher psychological distress, such as anxiety and depression, which may lead to lower smoking cessation success.^2^ Socially, financial hardship may be more strongly associated with difficulty in quitting smoking among women than among men.^9^ Finally, sex disparities in health care access can complicate smoking cessation efforts, particularly among women.^3^

Tobacco smoking is the leading preventable cause of lung cancer, accounting for up to 80% of cases.^10^ Hence, several countries, such as Canada, Croatia, South Korea, the United Kingdom, and the United States, have implemented lung cancer screening programs using low-dose computed tomography (LDCT) for high-risk individuals with at least a 20 pack-year smoking history, including persons with active tobacco use and former persons who smoke (PWS).^11^ LDCT screening has been associated with a 21% reduction in lung cancer-related mortality compared with chest radiography or no screening.^12^ In addition, LDCT screening often serves as a motivator for smoking cessation, with many participants citing their screening experience as a catalyst for quitting.^13^ Furthermore, combining LDCT with smoking cessation programs enhances cost-effectiveness.^14^

After a lung cancer diagnosis, smoking cessation has also been found to substantially reduce lung cancer-related mortality and all-cause mortality.^15^ However, despite its importance, limited research exists on smoking cessation interventions for people with lung cancer.^16^ Specifically, there remains a lack of targeted research addressing the sex-specific challenges of smoking cessation among individuals with lung cancer. The present study aims to address this gap by leveraging data from the French national database of smoking cessation services (SCS), Consultations de Dépendance Tabagique (CDTnet), to identify sex-specific factors influencing smoking cessation in people with lung cancer. Our study provides actionable insights to improve cessation outcomes, especially in the context of the ongoing lung cancer screening programs worldwide.

## Methods

### Study Design and Data Source

This retrospective observational study used data from CDTnet, the French national database of SCS (www.cdtnet.fr). Data were collected from 72 SCS that contributed continuously to CDTnet between January 1, 2001 and December 31, 2018. Most SCS were located in public hospitals (96.3%), with smaller proportions in addiction centers (1.3%), prison health services (1.2%), private hospitals (0.8%), general practitioners' offices (0.3%), and medical dispensaries (0.2%). At SCS, PWS seeking assistance in quitting are offered behavioral support and pharmacologic treatment such as nicotine replacement therapy (NRT), varenicline, or a combination of NRT and varenicline. The initial consultation lasts between 45 and 60 minutes, with follow-up consultations lasting approximately 30 minutes.

During their first consultation at a SCS, PWS were invited to complete a standardized paper questionnaire, developed since 2000 by a national working group under the initiative of Santé Publique France. The information provided was verified by the SCS staff and recorded in the CDTnet database after obtaining the individual's consent, which was documented by means of the paper questionnaire. Individuals included in the database were followed throughout their quit attempt, with smoking status systematically recorded at each consultation.

All data in CDTnet are anonymized. The CDTnet database is authorized by the French Data Protection Authority (Commission Nationale de l'Informatique et des Libertés) (authorization number 739406). All individuals included in the database provided consent before their data were collected and recorded in CDTnet. This study complies with the guidelines of the Declaration of Helsinki. As CDTnet data are anonymized and do not contain addresses or phone numbers, approval from an institutional review board or an independent local ethics committee is not required.

### Study Population

Adults (≥18 years old) registered in CDTnet from 2001 to 2018 were eligible for inclusion if they were actively using tobacco at the time of their first SCS consultation and reported a diagnosis of lung cancer. Active tobacco use was defined as self-reported smoking of combustible tobacco (cigarillos, rolled cigarettes, or manufactured cigarettes) every day or some days at the first consultation.^17^ To ensure sufficient follow-up, we included only participants with at least one follow-up SCS consultation after the initial consultation, as 50% of PWS are lost to follow-up after an initial contact in SCS.^18^ Consequently, we considered only PWS who attended at least one follow-up consultation in SCS for this study. Pregnant women were excluded. Among 6694 adult PWS with lung cancer registered in the CDTnet database between 2001 and 2018, 3407 adult PWS (50.9%) attended at least 2 SCS consultations and were hence included in the present analysis. Of these, 1063 adult PWS (31.2%) declared they were of female sex, and 2344 adult PWS (68.8%) declared they were male.

### Data

All data provided by PWS during their first SCS consultation were verified by SCS staff before being recorded in CDTnet. Sociodemographic variables collected included sex, age, employment status (employed, unemployed, or retired), and educational level (no diploma, vocational technical qualification, high school diploma, or higher education). Information on home smoking environment was also recorded, specifically whether individuals smoked indoors and whether they lived with other PWS.

Moreover, we collected information on self-reported medical history, including cardiovascular risk factors (hypertension, diabetes, or hypercholesterolemia), cardiovascular diseases (myocardial infarction, angina, stroke, or peripheral arterial disease), respiratory diseases (chronic obstructive pulmonary disease, chronic bronchitis, or asthma), and smoking-related cancers aside from lung cancer (i.e., head and neck or bladder cancer). Psychological data included a history of depression, presence of anxiety or depression symptoms, and psychotropic medication use. The Hospital Anxiety and Depression (HAD) scale was used to assess anxiety and depressive disorders. A score greater than 10 on the HAD-anxiety or HAD-depression subscale was used to indicate a clinically significant anxiety or depressive state, respectively.^19^

To assess the smoking behaviors of PWS registered in CDTnet, we gathered information on the reason for visiting a SCS (personal initiative, referral after hospital care, referral by a primary care health professional, or encouragement by an acquaintance), the number of previous quit attempts lasting 7 days or more, weight gain during past quit attempts, cigarette consumption reduction in the past month, daily smoking status, and the number of cigarettes smoked per day. A cigarillo was considered equivalent to two manufactured cigarettes, and a rolled cigarette was considered equivalent to two manufactured cigarettes.^20^ Nicotine dependence was assessed using the heaviness of smoking index (HSI), with a score of at least four indicating high dependence.^21^ The HSI is a self-report measure derived from the Fagerström test for nicotine dependence, including two questions: number of cigarettes smoked daily and time from waking up to the first cigarette of the day.^21^ Confidence in the ability to quit was measured using a visual analog scale (0–10), with high confidence defined as a score greater than or equal to seven. Co-addictions recorded included alcohol overuse (defined as the consumption of ≥2 alcoholic drinks per day) and cannabis use in the past 30 days.^22^ Dual use of conventional cigarettes and electronic cigarettes (with or without nicotine) has been recorded since October 2015.

Furthermore, smoking cessation treatments, including cognitive behavioral techniques, NRT, and varenicline, prescribed during the initial consultation at the SCS, were documented in CDTnet. The number of SCS consultations was also collected and classified as one to three, four to six, or 7 or more.

### Study Outcomes

The primary outcome was 28-day smoking abstinence, defined as self-reported smoking cessation for at least 28 consecutive days during follow-up, confirmed by an exhaled carbon monoxide (CO) measurement less than 10 parts per million at each follow-up consultation.^23^ This time frame was chosen on the basis of the criteria used for evaluating the effectiveness of English SCS, as most people who smoke relapse within the first few days of a serious quit attempt, and the likelihood of 1-year cessation increases fivefold after the first 4 weeks. Thus, 28-day abstinence is considered a reliable indicator of long-term cessation.^24^ The secondary outcome was a reduction in smoking, defined as at least a 50% decrease in cigarette consumption from the first consultation.^25^

### Statistical Analysis

All analyses were performed stratified by sex. Data distribution was assessed using D'Agostino's K-square test. Continuous variables were analyzed using either a Student’s *t* test or a Mann-Whitney U test, with results presented as mean (± SD). Categorical variables were analyzed using the chi-square test, with results presented as numbers and percentages. To identify factors associated with smoking abstinence, a univariate logistic regression, a multivariate logistic regression, and a stepwise multivariate logistic regression were performed. The dependent variable was abstinence, with participants classified as “yes” if they achieved the primary outcome and “no” if they either reduced smoking by at least 50% or continued smoking.

For the CO measurement, which had 11% missing data, multiple imputation was performed. Missing data for other variables was not identified. Two-tailed *p* values less than 0.05 and confidence intervals (CIs) of odds ratios (ORs) not inclusive of 1 were considered statistically significant. All statistical analyses were performed using R (version 4.2.1; R Foundation for Statistical Computing, Vienna, Austria).

## Results

### Overall Characteristics of the Study Population

Characteristics of the study population are presented in Table 1. Among 3407 PWS with lung cancer included in this analysis between 2001 and 2018, the mean (± SD) age was 56 (± 9) years, with men (n = 2344) being older than women (n = 1063) (57 versus 55 y; *p* < 0.001). Educational attainment differed significantly by sex (*p* < 0.001), as a higher proportion of men had vocational technical qualifications (36.7% versus 22.4%), whereas more women had no diploma (36.4% versus 32.0%) or higher education (21.4% versus 16.4%). Women were more often unemployed than men (34.5% versus 30.4%; *p* = 0.004).

**Table 1:** Baseline Characteristics in 3407 Smoking Individuals With Lung Cancer Followed-up in French SCS Between 2001 and 2018, According to Sex

Characteristics	Overall N = 3407	Women N = 1063	Men N = 2344	*p* Value
**Mean** ± **SD age (y)**	56 ± 9	55 ± 9	57 ± 8	**<0.001**
**Age groups (y), n (%)**				**0.004**
18–39	89 (2.6)	39 (3.7)	50 (2.1)	
40–49	616 (18.1)	218 (20.5)	398 (17.0)	
50–59	1560 (45.8)	478 (45.0)	1082 (46.2)	
60–69	929 (27.3)	267 (25.1)	662 (28.2)	
≥70	213 (6.3)	61 (5.7)	152 (6.5)	
**Level of education, n (%)**				**<0.001**
No diploma	1138 (33.4)	387 (36.4)	751 (32.0)	
Vocational technical qualification	1098 (32.2)	238 (22.4)	860 (36.7)	
High school diploma	559 (16.4)	211 (19.8)	348 (14.8)	
Higher education	612 (18.0)	227 (21.4)	385 (16.4)	
**Employment status, n (%)**				**0.004**
Employed	1359 (39.9)	431 (40.6)	928 (39.6)	
Unemployed	1079 (31.7)	367 (34.5)	712 (30.4)	
Retired	969 (28.4)	265 (24.9)	704 (30.0)	
**Home smoking environment, n (%)**				
Smokes at home	217 (6.4)	82 (7.7)	135 (5.8)	**0.03**
Other persons who smoke at home	103 (3.0)	39 (3.7)	64 (2.7)	0.14
**Cardiovascular risk factors or diseases, n (%)**				
Hypertension	621 (18.2)	178 (16.7)	443 (18.9)	0.20
Diabetes	220 (6.5)	57 (5.4)	163 (7.0)	0.73
Hypercholesterolemia	560 (16.4)	179 (16.8)	381 (16.3)	0.60
Myocardial infarction or angina	278 (8.2)	49 (4.6)	229 (9.8)	**<0.001**
Stroke	138 (4.1)	37 (3.5)	101 (4.3)	0.30
Peripheral arterial disease	339 (10.0)	51 (4.8)	288 (12.3)	**<0.001**
**Respiratory diseases, n (%)**				
Asthma	259 (7.6)	122 (11.5)	137 (5.8)	**<0.001**
Chronic bronchitis or COPD	929 (27.3)	324 (30.5)	605 (25.8)	**0.005**
**Other smoking-related cancers, n (%)**				
Bladder cancer	9 (0.3)	2 (0.2)	7 (0.3)	0.70
Head and neck cancer	17 (0.5)	2 (0.2)	15 (0.6)	0.83
**Psychological disorders, n (%)**				
Depression history	924 (27.1)	400 (37.6)	524 (22.4)	**<0.001**
Depression symptoms	519 (15.2)	211 (19.8)	308 (13.1)	**<0.001**
Anxiety symptoms	1097 (32.2)	457 (43.0)	640 (27.3)	**<0.001**
**Psychotropic medication use, n (%)**				
Anxiolytics	1005 (29.5)	402 (37.8)	603 (25.7)	**<0.001**
Antidepressants	784 (23.0)	355 (33.4)	429 (18.3)	**<0.001**
**Opioid substitution treatment, n (%)**	68 (2.0)	20 (1.9)	48 (2.0)	0.70
**Reason for consulting a SCS, n (%)**				**<0.001**
Personal initiative	525 (15.4)	206 (19.4)	319 (13.6)	
Referral after hospital care	2490 (73.1)	727 (68.4)	1763 (75.2)	
Referral by a primary care health professional	297 (8.7)	99 (9.3)	198 (8.4)	
Encouragement by an acquaintance	95 (2.8)	31 (2.9)	64 (2.7)	
**Previous attempts to quit lasting ≥7 days, n (%)**				**0.008**
0	1352 (39.7)	409 (38.5)	943 (40.2)	
1–2	1562 (45.8)	470 (44.2)	1092 (46.6)	
≥3	493 (14.5)	184 (17.3)	309 (13.2)	
**Weight gain during previous quit attempts, n (%)**	40 (1.2)	18 (1.7)	22 (0.9)	0.058
**Cigarette consumption reduction in past month, n (%)**	128 (3.8)	41 (3.9)	87 (3.7)	0.80
**Smokes every day, n (%)**	2647 (77.7)	822 (77.3)	1825 (77.9)	0.70
**Mean** ± **SD number of cigarettes smoked daily**	26 ± 18	25 ± 18	27 ± 19	**0.002**
**Number of cigarettes smoked daily, n (%)**				**<0.001**
≤10	673 (19.8)	218 (20.5)	455 (19.4)	
11–20	1211 (35.5)	414 (38.9)	797 (34.0)	
21–40	1153 (33.8)	343 (32.3)	810 (34.6)	
≥41	370 (10.9)	88 (8.3)	282 (12.0)	
**Time from waking up to first cigarette, n (%)**				0.70
Within 5 min	793 (23.3)	244 (23.0)	549 (23.4)	
6–30 min	298 (8.7)	92 (8.7)	206 (8.8)	
31–60 min	1071 (31.4)	323 (30.4)	748 (31.9)	
Over 60 min	1245 (36.5)	404 (38.0)	841 (35.9)	
**Nicotine dependence assessed by HSI, n (%)**				0.50
Low: 0–1	296 (8.7)	100 (9.4)	196 (8.4)	
Moderate: 2–3	936 (27.5)	283 (26.6)	653 (27.9)	
High: 4–6	2175 (63.8)	680 (64.0)	1495 (63.8)	
**Confidence in ability to quit, n (%)**				0.05
Low: 0–4	1486 (43.6)	492 (46.3)	994 (42.4)	
Moderate: 5–6	926 (27.2)	288 (27.1)	638 (27.2)	
High: 7–10	995 (29.2)	283 (26.6)	712 (30.4)	
**Other addictive substances, n (%)**				
≥2 glasses of alcohol per day	985 (28.9)	148 (13.9)	837 (35.7)	**<0.001**
Cannabis consumption in the past 30 d	182 (5.3)	58 (5.5)	124 (5.3)	0.80
Dual use of electronic and conventional cigarettes	109 (3.2)	41 (3.9)	68 (2.9)	0.14
**Smoking cessation treatments prescribed at the first SCS consultation, n (%)**				**0.015**
No pharmacotherapy (CBT)	466 (13.7)	174 (16.4)	292 (12.5)	
NRT +CBT	2808 (82.4)	852 (80.2)	1956 (83.4)	
Varenicline + CBT	110 (3.2)	28 (2.6)	82 (3.5)	
Varenicline + NRT + CBT	23 (0.7)	9 (0.8)	14 (0.6)	
**Mean** ± **SD number of follow-up consultations**	3 ± 4	4 ± 4	3 ± 4	**0.001**
**Number of follow-up consultations, n (%)**				**0.002**
1–3	2464 (72.3)	726 (68.3)	1738 (74.1)	
4–6	571 (16.8)	197 (18.5)	374 (16.0)	
≥7	372 (10.9)	140 (13.2)	232 (9.9)	
**28-day smoking abstinence outcome, n (%)**				0.40
Abstinence	1203 (35.3)	374 (35.2)	829 (35.4)	
Reduction	1082 (31.8)	353 (33.2)	729 (31.1)	
Persistent smoking	1122 (32.9)	336 (31.6)	786 (33.5)	

*p* Values less than 0.05 denote statistically significant differences between men and women.

Bold values represent statistically significant results.

CBT, cognitive behavioral techniques; COPD, chronic obstructive pulmonary disease; HSI, heaviness of smoking index; NRT, Nicotine replacement therapy; SCS, smoking cessation services.

In terms of health, men reported higher rates of cardiovascular conditions—myocardial infarction or angina was present in 9.8% of men compared with 4.6% of women (*p* < 0.001), and peripheral arterial disease in 12.3% versus 4.8% (*p* < 0.001). By contrast, respiratory conditions such as asthma were more prevalent among women (11.5% versus 5.8%; *p* < 0.001), as was chronic bronchitis or chronic obstructive pulmonary disease (30.5% versus 25.8%; *p* = 0.005). Psychological distress and psychotropic medication intake were significantly higher in women, with 37.6% reporting a history of depression compared with 22.4% of men, and anxiety symptoms noted in 43.0% versus 27.3% (*p* < 0.001 for both).

Smoking patterns were overall similar between sexes, with approximately 77% of both women and men smoking daily (*p* = 0.70). However, men smoked more cigarettes per day (mean ± SD of 27 ± 19) than women (25 ± 18; *p* = 0.002). Men were also significantly more likely to consume at least two alcoholic drinks per day (35.7% versus 13.9%; *p* < 0.001). Women were more likely than men to personally initiate consultations to SCS (19.4% versus 13.6%; *p* < 0.001). In addition, women, on average, attended more follow-up consultations at a SCS than men (4 ± 4 versus 3 ± 4; *p* = 0.001). Despite these differences, the 28-day smoking abstinence rate was nearly identical between women (35.2%) and men (35.4%; *p* = 0.40). There was also no difference in smoking reduction between women and men, with women exhibiting a reduction of 33.2% and men 31.1%.

### Characteristics of the Study Population Stratified by Smoking Abstinence Outcomes

Characteristics of the study population, stratified by 28-day smoking abstinence outcomes (no abstinence, abstinence, or smoking reduction), are presented in Table 2. Among women, those who were employed had a higher proportion of 28-day smoking abstinence (41.3% employed versus 30.4% for those who did not quit smoking; *p* = 0.009). Among men, employment also correlated with higher abstinence rates (37.6% employed versus 26.7% for those who reduced smoking; *p* < 0.001). Both women and men who smoked every day had a significantly lower likelihood of quitting (*p* < 0.001). High confidence in the ability to quit was also a significant factor for smoking abstinence in both sexes (*p* < 0.001). Furthermore, follow-up SCS consultations were significantly more frequent among those who achieved smoking abstinence. Both men and women who quit smoking attended a mean (± SD) of 5 (± 6) consultations, compared with 2 (± 2) consultations in women and 2 (± 1) consultations in men among those who did not quit, and 3 (± 4) consultations among those who reduced smoking (*p* < 0.001).

**Table 2:** Characteristics of Patients With Lung Cancer Followed-up in French SCS Between 2001 and 2018, Categorized by 28-day Smoking Abstinence Outcome

Characteristics	Women				Men			
	No N = 336	Yes N = 374	Reduction N = 353	*p*-value	No N = 786	Yes N = 829	Reduction N = 729	*p*-value
**Mean** ± **SD age (y)**	55 ± 9	55 ± 9	56 ± 9	0.80	56 ± 9	57 ± 8	57 ± 8	0.70
**Age groups, n (%)**				0.70				0.20
18–39	15/39 (38.5)	16/39 (41.0)	8/39 (20.5)		20/50 (40.0)	15/50 (30.0)	15/50 (30.0)	
40–49	64/218 (29.4)	79/218 (36.2)	75/218 (34.4)		148/398 (37.2)	133/398 (33.4)	117/398 (29.4)	
50–59	157/478 (32.8)	165/478 (34.5)	156/478 (32.6)		362/1,082 (33.5)	384/1,082 (35.5)	336/1,082 (31.0)	
60–69	79/267 (39.6)	96/267 (36.0)	92/267 (34.4)		206/662 (31.1)	244/662 (36.9)	212/662 (32.0)	
≥70	21/61 (34.4)	18/61 (29.5)	22/61 (36.1)		50/152 (32.9)	53/152 (35.9)	49/152 (32.2)	
**Education level, n (%)**				0.60				0.30
No diploma	132/387 (34.1)	142/387 (36.7)	113/387 (29.2)		257/751 (34.2)	279/751 (37.2)	215/751 (28.6)	
Vocational	73/238 (30.7)	81/238 (34.0)	84/238 (35.3)		271/860 (31.5)	304/860 (35.3)	285/860 (33.2)	
High school diploma	63/211 (29.9)	71/211 (33.6)	77/211 (36.5)		114/348 (32.8)	119/348 (34.2)	115/348 (33.0)	
Higher education	68/227 (30.0)	80/227 (35.2)	79/227 (34.8)		144/385 (37.4)	127/385 (33.0)	114/385 (29.6)	
**Employment, n (%)**				**0.009**				**<0.001**
Employed	131/431 (30.4)	178/431 (41.3)	122/431 (28.3)		331/928 (35.7)	349/928 (37.6)	248/928 (26.7)	
Unemployed	119/367 (32.4)	111/367 (30.2)	137/367 (37.4)		232/712 (32.6)	223/712 (31.3)	257/712 (36.1)	
Retired	86/265 (32.5)	85/265 (32.1)	94/265 (35.5)		223/704 (31.7)	257/704 (36.5)	224/704 (31.8)	
**Home smoking environment, n (%)**								
Smokes at home	25/82 (30.5)	29/82 (35.4)	28/82 (34.1)	>0.90	38/135 (28.1)	50/135 (37.0)	47/135 (34.8)	0.40
Other PWS at home	13/39 (33.3)	19/39 (48.7)	7/39 (17.9)	0.083	22/64 (34.4)	23/64 (35.9)	19/64 (29.7)	>0.90
**Cardiovascular risk factors or diseases, n (%)**								
Hypertension	54/178 (30.3)	65/178 (36.5)	59/178 (33.1)	0.90	160/443 (36.1)	140/443 (31.6)	143/443 (32.3)	0.20
Diabetes	18/57 (31.6)	22/57 (38.6)	17/57 (29.8)	0.80	59/163 (36.2)	51/163 (31.3)	53/163 (32.5)	0.50
Hypercholesterolemia	52/179 (29.1)	65/179 (36.3)	62/179 (34.6)	0.70	125/381 (32.8)	135/381 (35.4)	121/381 (31.8)	>0.90
MI or angina	13/49 (26.5)	22/49 (44.9)	14/49 (28.6)	0.30	72/229 (31.4)	77/229 (33.6)	80/229 (34.9)	0.40
Stroke	14/37 (37.8)	14/37 (37.8)	9/37 (24.3)	0.50	38/101 (37.6)	28/101 (27.7)	35/101 (34.7)	0.30
PAD	16/51 (31.4)	19/51 (37.3)	16/51 (31.4)	>0.90	97/288 (33.7)	96/288 (33.3)	95/288 (33.0)	0.70
**Respiratory diseases, n (%)**								
Asthma	43/122 (35.2)	39/122 (32.0)	40/122 (32.8)	0.60	40/137 (29.2)	52/137 (38.0)	45/137 (32.8)	0.50
Chronic bronchitis or COPD	106/324 (32.7)	100/324 (30.9)	118/324 (36.4)	0.13	194/605 (32.1)	215/605 (35.5)	196/605 (32.4)	0.60
**Other smoking-related cancers, n (%)**								
Bladder cancer	0/2 (0.0)	1/2 (50.0)	1/2 (50.0)	>0.90	2/7 (28.6)	3/7 (42.9)	2/7 (28.5)	>0.90
Head and neck cancer	1/2 (50.0)	0/2 (0.0)	1/2 (50.0)	0.50	3/15 (20.0)	6/15 (40.0)	6/15 (40.0)	0.50
**Psychological disorders, n (%)**								
Depression history	132/400 (33.0)	126/400 (31.5)	142/400 (35.5)	0.14	175/524 (33.4)	185/524 (35.3)	164/524 (31.3)	>0.90
Depression symptoms	73/211 (34.6)	65/211 (30.8)	73/211 (34.6)	0.31	101/308 (32.8)	100/308 (32.5)	107/308 (34.7)	0.30
Anxiety symptoms	145/457 (31.7)	157/457 (34.4)	155/457 (33.9)	0.90	203/640 (31.7)	229/640 (35.8)	208/640 (32.5)	0.50
**Psychotropic medication use, n (%)**								
Anxiolytics	134/402 (33.3)	126/402 (31.3)	142/402 (35.3)	0.12	198/603 (32.8)	211/603 (35.0)	194/603 (32.2)	0.80
Antidepressants	115/355 (32.4)	109/355 (30.7)	131/355 (36.9)	0.069	135/429 (31.5)	144/429 (33.5)	150/429 (35.0)	0.20
**OST, n (%)**	5/20 (25.0)	9/20 (45.0)	6/20 (30.0)	0.60	17/48 (35.4)	9/48 (18.8)	22/48 (45.8)	**0.026**
**Reason for consulting a SCS, n (%)**				0.13				**0.004**
Personal initiative	63/206 (30.6)	68/206 (33.0)	75/206 (36.4)		101/319 (31.7)	111/319 (34.8)	107/319 (33.5)	
Hospital care	230/727 (31.6)	272/727 (37.4)	225/727 (31.0)		605/1,763 (34.3)	630/1,763 (35.7)	528/1,763 (30.0)	
Referral by health professionals	31/99 (31.3)	25/99 (25.3)	43/99 (43.4)		54/198 (27.3)	61/198 (30.8)	83/198 (41.9)	
Encouraged by acquaintances	12/31 (38.7)	9/31 (29.0)	10/31 (32.3)		26/64 (40.6)	27/64 (42.2)	11/64 (17.2)	
**Previous attempts to quit lasting ≥7 days, n (%)**				0.80				0.70
0	128/409 (31.3)	144/409 (35.2)	137/409 (33.5)		302/943 (32.0)	340/943 (36.1)	301/943 (31.9)	
1–2	145/470 (30.9)	163/470 (34.7)	162/470 (34.5)		382/1,092 (35.0)	379/1,092 (34.7)	331/1,092 (30.3)	
≥3	63/184 (34.2)	67/184 (36.4)	54/184 (29.4)		102/309 (33.0)	110/309 (35.6)	97/309 (31.4)	
**Weight gain in past quit attempts, n (%)**	8/18 (44.4)	7/18 (38.9)	3/18 (16.7)	0.30	6/22 (27.3)	10/22 (45.5)	6/22 (27.3)	0.60
**Cigarette reduction in past month, n (%)**	13/41 (31.7)	16/41 (39.0)	12/41 (29.3)	0.80	26/87 (29.9)	33/87 (37.9)	28/87 (32.2)	0.80
**Smokes daily, n (%)**	238/822 (29.0)	253/822 (30.8)	331/822 (40.3)	**<0.001**	556/1,825 (30.5)	574/1,825 (31.5)	695/1,825 (38.1)	**<0.001**
**Mean** ± **SD number of cigarettes smoked daily**	25 ± 17	25 ± 17	25 ± 18	>0.90	26 ± 18	27 ± 17	27 ± 21	0.30
**Number of cigarettes smoked daily, n (%)**				>0.90				0.20
≤10	68/218 (31.2)	80/218 (36.7)	70/218 (32.1)		159/455 (34.9)	140/455 (30.8)	156/455 (34.3)	
11–20	130/414 (31.4)	141/414 (34.1)	143/414 (34.5)		267/797 (33.5)	283/797 (35.5)	247/797 (31.0)	
21–40	112/343 (32.7)	119/343 (34.7)	112/343 (32.7)		271/810 (33.5)	307/810 (37.9)	232/810 (28.6)	
≥41	26/88 (29.5)	34/88 (38.6)	28/88 (31.9)		89/282 (31.6)	99/282 (35.1)	94/282 (33.3)	
**Time from waking up to first cigarette, n (%)**				**<0.001**				**<0.001**
Within 5 min	100/244 (41.0)	119/244 (48.8)	25/244 (10.2)		242/549 (44.1)	257/549 (46.8)	50/549 (9.1)	
6–30 min	19/92 (20.7)	41/92 (44.6)	32/92 (34.8)		65/206 (31.6)	71/206 (34.4)	70/206 (34.0)	
31–60 min	93/323 (28.8)	106/323 (32.8)	124/323 (38.4)		215/748 (28.7)	254/748 (34.0)	279/748 (37.3)	
Over 60 min	124/404 (30.7)	108/404 (26.7)	172/404 (42.6)		264/841 (31.4)	247/841 (29.4)	330/841 (39.2)	
**Nicotine dependence assessed by HSI, n (%)**				**0.010**				0.60
Low: 0–1	33/100 (33.0)	45/100 (45.0)	22/100 (22.0)		60/196 (30.6)	76/196 (38.8)	60/196 (30.6)	
Moderate: 2–3	83/283 (29.3)	113/283 (39.9)	87/283 (30.7)		228/653 (34.9)	234/653 (35.8)	191/653 (29.2)	
High: 4–6	220/680 (32.4)	216/680 (31.8)	244/680 (35.9)		498/1,495 (33.3)	519/1,495 (34.7)	478/1,495 (32.0)	
**Confidence in ability to quit, n (%)**				**<0.001**				**<0.001**
Low: 0–4	176/492 (35.8)	179/492 (36.4)	137/492 (27.8)		363/994 (36.5)	381/994 (38.3)	250/994 (25.2)	
Moderate: 5–6	78/288 (27.1)	88/288 (30.6)	122/288 (42.4)		199/638 (31.2)	187/638 (29.3)	252/638 (39.5)	
High: 7–10	82/283 (29.0)	107/283 (37.8)	94/283 (33.2)		224/712 (31.5)	261/712 (36.6)	227/712 (31.9)	
**Other addictive substances, n (%)**								
≥2 alcohol glasses/day	42/148 (28.4)	51/148 (34.4)	55/148 (37.2)	0.50	296/837 (35.4)	290/837 (34.6)	251/837 (30.0)	0.40
Cannabis use in past 30 days	21/58 (36.2)	16/58 (27.6)	21/58 (36.2)	0.50	47/124 (37.9)	38/124 (30.6)	39/124 (31.5)	0.50
Dual users of electronic and conventional cigarettes	13/41 (31.7)	17/41 (41.5)	11/41 (26.8)	0.60	17/68 (25.0)	29/68 (42.6)	22/68 (32.4)	0.30
**Smoking cessation treatment prescribed at first consultation, n (%)**				**0.02**				**0.03**
No pharmacotherapy (CBT)	63/174 (36.2)	66/174 (37.9)	45/174 (25.9)		118/292 (40.4)	85/292 (29.1)	89/292 (30.5)	
NRT + CBT	264/852 (31.0)	293/852 (34.4)	295/852 (34.6)		644/1,956 (32.9)	713/1,956 (36.5)	599/1,956 (30.6)	
Varenicline + CBT	5/28 (17.9)	12/28 (42.9)	11/28 (39.3)		23/82 (28.0)	25/82 (30.5)	34/82 (41.5)	
Varenicline + NRT + CBT	4/9 (44.4)	3/9 (33.3)	2/9 (22.2)		1/14 (7.1)	6/14 (42.9)	7/14 (50.0)	
**Mean** ± **SD number of follow-up consultations**	2 ± 2	5 ± 6	3 ± 4	**<0.001**	2 ± 1	5 ± 6	3 ± 4	**<0.001**
**Number of follow-up consultations, n (%)**				**<0.001**				**<0.001**
1–3	307/726 (42.3)	175/726 (24.1)	244/726 (33.6)		726/1,738 (41.8)	477/1,738 (27.4)	535/1,738 (30.8)	
4–6	23/197 (11.7)	108/197 (54.8)	66/197 (33.5)		50/374 (13.4)	194/374 (51.9)	130/374 (34.8)	
≥7	6/140 (4.3)	91/140 (65.0)	43/140 (30.7)		10/232 (4.3)	158/232 (68.1)	64/232 (27.6)	

The *p* Values less than 0.05 denote statistically significant differences.

Bold values represent statistically significant results.

CBT, cognitive behavioral techniques; COPD, chronic obstructive pulmonary disease; HSI, Heaviness of Smoking Index; MI, myocardial infarction; NRT, nicotine replacement therapy; OST, opioid substitution therapy; PAD, peripheral artery disease; PWS, persons who smoke; SCS, smoking cessation service.

### Predictors of Smoking Cessation

Multivariate logistic regression analyses revealed both common and sex-specific predictors of 28-day smoking abstinence (Table 3). Confidence in quitting was a strong predictor of smoking cessation in both men and women. Individuals with high confidence had significantly increased odds of achieving abstinence (women: OR = 1.94; 95% CI: 1.30–2.92; *p* = 0.001; men: OR = 1.50; 95% CI: 1.16–1.95; *p* = 0.002). Follow-up consultations were another key factor for both sexes, with a dose-response relationship observed. Attending 4 to 6 SCS consultations was associated with an OR of 4.93 in women (95% CI: 3.41–7.17; *p* < 0.001) and 3.20 in men (95% CI: 2.51–4.07; *p* < 0.001), whereas individuals with 7 or more consultations had an OR of 8.87 in women (95% CI: 5.70–14.0; *p* < 0.001) and 6.66 in men (95% CI: 4.89–9.16; *p* < 0.001). Daily smoking was a common barrier to cessation in both sexes. Women who smoked every day had lower odds of quitting (OR = 0.32; 95% CI: 0.17–0.58; *p* < 0.001), and a similar association was found in men (OR = 0.43; 95% CI: 0.29–0.63; *p* < 0.001).

**Table 3:** Multivariate Analysis of Factors Associated With Smoking Cessation Among Individuals With Lung Cancer Followed-up in French SCS Between 2001 and 2018, Stratified by Sex

Characteristics	Women			Men		
	OR	95% CI	*p*-value	OR	95% CI	*p* Value
**Age groups (y)**						
18–39	Reference					
40–49	0.83	0.36–1.96	0.66	0.95	0.48–1.94	0.88
50–59	0.84	0.37–1.94	0.67	1.18	0.61–2.38	0.62
60–69	1.17	0.48–2.92	0.73	1.34	0.66–2.82	0.43
≥70	0.90	0.30–2.71	0.85	1.44	0.64–3.33	0.38
**Level of education**						
No diploma	Reference					
Vocational qualification	0.82	0.55–1.23	0.35	0.87	0.70–1.10	0.25
High school diploma	0.80	0.52–1.21	0.29	0.86	0.64–1.15	0.32
Higher education	0.75	0.49–1.14	0.19	0.76	0.56–1.01	0.061
**Employment status**						
Employed	Reference					
Unemployed	0.71	0.50–1.00	0.054	**0.73**	**0.58–0.93**	**0.009**
Retired	**0.52**	**0.31–0.87**	**0.013**	0.89	0.64–1.23	0.47
**Home smoking environment**						
Smokes at home	0.67	0.34–1.32	0.26	1.02	0.62–1.64	0.95
Other PWS at home	**4.03**	**1.70–10.0**	**0.002**	0.92	0.48–1.73	0.79
**Cardiovascular risk factors or diseases**						
Hypertension	0.99	0.65–1.51	0.97	0.89	0.69–1.15	0.38
Diabetes	1.06	0.53–2.09	0.86	0.94	0.64–1.37	0.76
Hypercholesterolemia	1.20	0.79–1.82	0.40	1.03	0.78–1.34	0.85
Myocardial infarction or angina	1.48	0.74–2.98	0.26	0.91	0.65–1.27	0.59
Stroke	1.51	0.64–3.43	0.34	0.83	0.50–1.34	0.46
Peripheral artery disease	1.23	0.60–2.48	0.56	0.93	0.69–1.25	0.64
**Respiratory diseases**						
Asthma	0.77	0.46–1.29	0.33	1.07	0.71–1.60	0.74
Chronic bronchitis or COPD	0.76	0.53–1.08	0.13	0.96	0.77–1.20	0.73
**Other smoking-related cancers**						
Bladder cancer	0.63	0.02–20.2	0.77	1.96	0.35–9.62	0.41
Head and neck cancer	0.00		>0.90	1.35	0.39–4.30	0.61
**Psychological disorders**						
Depression history	0.81	0.57–1.14	0.23	0.98	0.76–1.26	0.89
Depression symptoms	0.88	0.59–1.32	0.55	0.89	0.66–1.20	0.45
Anxiety symptoms	1.00	0.72–1.38	0.98	1.09	0.87–1.36	0.47
**Psychotropic medication use**						
Anxiolytics	0.90	0.63–1.28	0.55	1.14	0.88–1.46	0.32
Antidepressants	0.77	0.53–1.13	0.19	0.88	0.65–1.18	0.39
**Opioid substitution treatment**	2.30	0.76–6.83	0.13	0.52	0.22–1.09	0.10
**Reason for consulting a SCS**						
Personal initiative	Reference					
Referral after hospital care	**1.63**	**1.10–2.43**	**0.016**	1.17	0.89–1.55	0.27
Referral by health professionals	0.83	0.44–1.58	0.59	0.79	0.52–1.20	0.28
Encouragement by acquaintances	1.22	0.45–3.09	0.69	1.42	0.78–2.56	0.24
**Previous attempts to quit lasting ≥7 days**						
0	Reference					
1–2	0.97	0.70–1.35	0.87	0.87	0.71–1.07	0.19
≥3	0.86	0.55–1.33	0.51	0.86	0.63–1.16	0.32
**Weight gain during past quit attempts**	1.17	0.33–3.91	0.80	1.36	0.50–3.65	0.54
**Cigarette consumption reduction in the past month**	0.66	0.26–1.62	0.40	1.06	0.61–1.80	0.80
**Smokes every day**	**0.32**	**0.17–0.58**	**<0.001**	**0.43**	**0.29–0.63**	**<0.001**
**Number of cigarettes smoked per day**						
≤10	Reference					
11–20	0.90	0.59–1.38	0.63	**1.43**	**1.08–1.90**	**0.012**
21–40	1.27	0.80–2.03	0.31	**1.90**	**1.40–2.57**	**<0.001**
≥41	1.76	0.91–3.38	0.091	**2.02**	**1.38–2.96**	**<0.001**
**Time from waking up to first cigarette**						
Within 5 min	Reference					
6–30 min	1.03	0.51–2.11	0.93	0.86	0.54–1.35	0.50
31–60 min	0.80	0.42–1.52	0.49	0.82	0.55–1.23	0.34
Over 60 min	0.65	0.34–1.24	0.19	0.67	0.44–1.03	0.065
**Nicotine dependence assessed by HSI**						
Low: 0–1	Reference					
Moderate: 2–3	1.07	0.60–1.92	0.82	0.84	0.57–1.25	0.39
High: 4–6	0.68	0.37–1.25	0.21	0.72	0.48–1.09	0.12
**Confidence in ability to quit**						
Low: 0–4	Reference					
Moderate: 5–6	1.25	0.83–1.89	0.28	1.07	0.82–1.41	0.62
High: 7–10	**1.94**	**1.30–2.92**	**0.001**	**1.50**	**1.16–1.95**	**0.002**
**Other addictive substances**						
≥2 glasses of alcohol per day	1.01	0.65–1.56	>0.90	0.89	0.73–1.08	0.25
Cannabis consumption in past 30 days	0.67	0.31–1.38	0.29	1.12	0.71–1.74	0.62
Dual users of electronic and conventional cigarettes	1.19	0.54–2.59	0.66	1.56	0.90–2.68	0.11
**Smoking cessation treatments prescribed at the first SCS consultation**						
No pharmacotherapy (CBT)	Reference					
NRT + CBT	0.97	0.64–1.47	0.88	**1.46**	**1.09–1.97**	**0.011**
Varenicline + CBT	1.39	0.51–3.69	0.52	1.35	0.75–2.38	0.31
Varenicline + NRT + CBT	1.08	0.17–5.69	>0.90	1.63	0.46–5.54	0.43
**Number of follow-up consultations**						
1–3	Reference					
4–6	**4.93**	**3.41–7.17**	**<0.001**	**3.20**	**2.51–4.07**	**<0.001**
≥7	**8.87**	**5.70–14.0**	**<0.001**	**6.66**	**4.89–9.16**	**<0.001**

Two-tailed *p* values less than 0.05 and CIs of ORs not inclusive of 1 were considered statistically significant.

Bold values represent statistically significant results.

CBT, cognitive behavioral techniques; CI, confidence interval; COPD, chronic obstructive pulmonary disease; HSI, heaviness of smoking index; NRT, nicotine replacement therapy; OR, odds ratio; PWS, persons who smoke.

**Figure 1:**
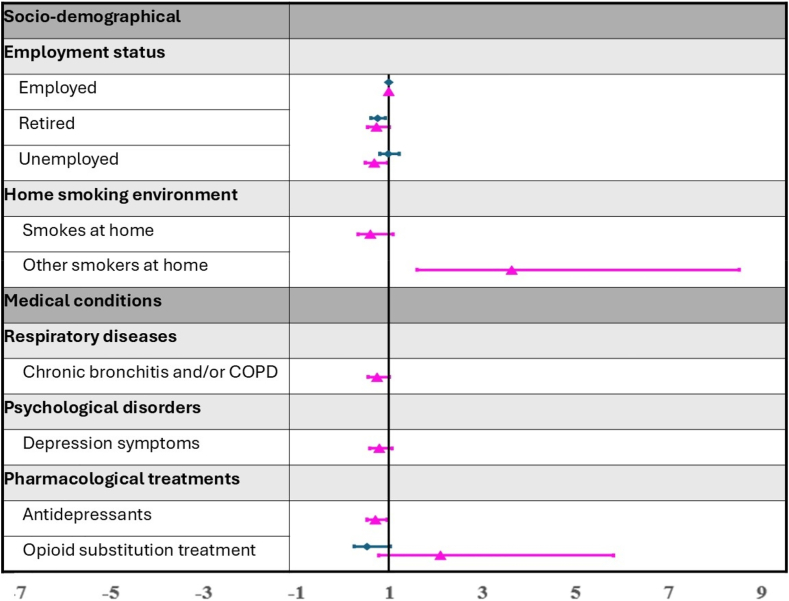
Forest plot of sociodemographic and medical factors associated with abstinence in women and men on the basis of stepwise multivariate analysis (adjusted odds ratio [95% confidence interval]), with blue representing men and pink representing women. COPD, chronic obstructive pulmonary disease.

**Figure 2:**
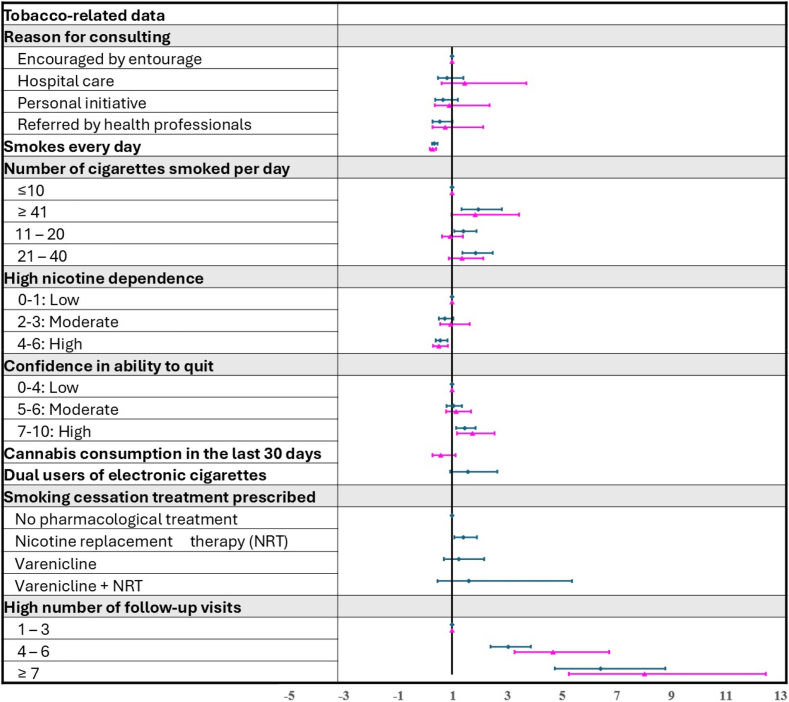
Forest plot of tobacco-related factors associated with abstinence in women and men on the basis of stepwise multivariate analysis (adjusted odds ratio [95% confidence interval]), with blue representing men and pink representing women.

Among women, referral after hospital care emerged as a facilitating factor, with these women more likely to achieve abstinence compared with women initiating cessation on their own (OR = 1.63; 95% CI: 1.10–2.43; *p* = 0.016). The presence of other PWS at home was also associated with an increased likelihood of smoking abstinence in women, with an OR of 4.03 (95% CI: 1.70–10.0; *p* = 0.002). Among men, employment status was a significant factor, with unemployed individuals having reduced odds of cessation compared with those who were employed (OR = 0.73; 95% CI: 0.58–0.93; *p* = 0.009). In addition, smoking intensity exhibited an inverse relationship with cessation success. Compared with those smoking 10 or fewer cigarettes per day, men who smoked 11 to 20 cigarettes had higher odds of quitting (OR = 1.43; 95% CI: 1.08–1.90; *p* = 0.012), with even greater odds for those smoking 21 to 40 cigarettes (OR = 1.90; 95% CI: 1.40–2.57; *p* < 0.001) and 41 or more cigarettes (OR = 2.02; 95% CI, 1.38–2.96; *p* < 0.001). NRT contributed positively to smoking abstinence, as men using NRT had greater odds of quitting (OR = 1.46; 95% CI: 1.09–1.97; *p* = 0.011). Figures 1 and 2 display the results of the stepwise multivariate analysis, illustrating sociodemographic, medical, and tobacco-related factors associated with smoking abstinence in women and men.

## Discussion

This longitudinal study identified sex-specific factors associated with smoking cessation among individuals with lung cancer seeking support from French SCS. Women, who comprised 31% of the study population, were more likely than men to be unemployed and to have either no formal education or higher education. They had lower rates of cardiovascular disease but were more frequently affected by respiratory diseases, experienced symptoms of depression and anxiety more often, and were more likely to use antidepressants and anxiolytics.

Despite notable differences in sociodemographic, health, and psychological characteristics, smoking abstinence rates at 28 days of follow-up were identical in men and women (35%). In addition, men in our study smoked more cigarettes per day and had made fewer previous attempts to quit smoking than women. Women were also more likely to seek smoking cessation consultations on their own initiative, whereas three-quarters of men were referred after hospitalization. Yet, men achieved similar smoking cessation and reduction rates as women, possibly reflecting a greater responsiveness to pharmacologic treatments such as NRT. Indeed, NRT contributed positively to smoking abstinence in men, as those using NRT had 1.46 times greater odds of quitting in multivariate logistic regression (95% CI: 1.09–1.97; *p* = 0.011). This was further confirmed in the stepwise multivariate analysis, in which NRT in men was associated with greater odds of quitting (OR = 1.43; 95% CI: 1.08–1.92; *p* = 0.015). Our finding is in line with previous studies, highlighting that NRT is less effective in women than in men.^26^^,^^27^ Women may be less responsive to NRT than men, as they metabolize nicotine more quickly and have higher nicotine clearance rates.^26^^,^^28^ Consequently, smoking behavior in women appears to be less reinforced by nicotine and more influenced by the sensory effects of smoking and psychological factors, such as social interaction and tension reduction, compared with men.^29^ This may partly explain why the presence of other PWS at home was associated with an increased likelihood of smoking abstinence in women, with an OR of 4.03 (95% CI: 1.70–10.0; p = 0.002) in multivariate analysis. This effect was also maintained in stepwise multivariate analysis (OR = 3.68; 95% CI: 1.59–8.68; *p* = 0.002). Interestingly, whereas social interactions generally encourage smoking, the presence of PWS at home might also motivate women to quit, either because of the repulsion of smoking behavior in close proximity or as a catalyst for making healthier lifestyle changes.^30^ These findings highlight the importance of active counseling in smoking cessation programs, in which social factors and personal motivations are considered to better support women in quitting.^30^^,^^31^

Indeed, for both women and men, a higher number of consultations was a strong predictor of smoking cessation. In men, the likelihood of smoking cessation tripled with 4 to 6 follow-up consultations and increased more than sixfold with 7 or more follow-up consultations. In women, the odds of quitting were even more pronounced, with an OR exceeding 4 for four to six follow-up consultations and exceeding 8 for seven or more follow-up consultations. Our data highlight the crucial role of follow-up consultations in smoking cessation, particularly for individuals with lower education levels and those who are unemployed—groups that are more likely to develop lung cancer than wealthier individuals who smoke and face greater challenges in quitting.^32^ These findings align with previous research, emphasizing the importance of intensive follow-up in smoking cessation programs for individuals with lung cancer.^31^^,^^33^ Counseling not only provides structured support but also enhances self-confidence in one's ability to quit smoking, which in turn, contributes to successful cessation.^34^ This was evident in our study, in which higher confidence in quitting was significantly associated with smoking cessation in both men (OR = 1.50; 95% CI: 1.16–1.95; *p* = 0.002) and women (OR = 1.94; 95% CI: 1.30–2.92; *p* = 0.001). These associations remained significant in stepwise multivariate analysis (women: OR = 1.79; 95% CI: 1.22–2.63; *p* = 0.003; men: OR = 1.47; 95% CI: 1.15–1.88; *p* = 0.002), reinforcing the role of self-efficacy as a key determinant of smoking cessation success.

Interestingly, in our study, men with high daily cigarette consumption were more likely to quit compared with those smoking 10 or fewer cigarettes per day. This contrasts with previous population-based studies in patients with cancer, in which smoking cessation was significantly associated with lower daily cigarette consumption.^33^^,^^35^ One plausible explanation is that individuals with high daily cigarette consumption may experience more severe cancer-related symptoms or treatment adverse effects, making smoking less tolerable and reinforcing cessation.^36^ In addition, they may receive stronger medical advice and more intensive cessation support from SCS health care providers. Overall, integrating smoking cessation support within oncology care—starting at the point of diagnosis and continuing throughout the cancer continuum—is essential for achieving the best success in quitting and maintaining lifelong abstinence.^33^

A major strength of this study is the use of data from CDTnet, the French national database of SCS, which provides a large and diverse real-world sample of persons with lung cancer who are supported in their attempt to quit smoking. The detailed sociodemographic, medical, and smoking behavior data allowed for robust analyses of sex-specific predictors of smoking cessation. In addition, the validation of the measure of exhaled CO less than 10 parts per million at each follow-up consultation enhanced the reliability of self-reported smoking abstinence. However, several limitations should be considered. The study population consisted of individuals who were mainly hospital-referred to SCS after a diagnosis of lung cancer, which may limit generalizability to individuals with lung cancer who do not engage with SCS. Moreover, whereas we controlled for multiple confounding factors, unmeasured variables—such as specific cancer treatments and their impact on cessation motivation, including lung cancer surgery—were not accounted for. A key limitation of this study is the lack of clinical data (e.g., cancer stage, diagnosis date, treatment status) in the CDTnet database, which restricts our ability to assess the influence of medical context on smoking cessation outcomes in people with lung cancer. Given that lung cancer is still often detected at an advanced stage,^37^ disease severity could influence motivation to quit smoking, either discouraging cessation attempts or, conversely, prompting individuals to quit to support their treatment. Smoking cessation is particularly relevant in this context, as it can reduce postoperative complications in surgical patients,^38^, ^39^, ^40^ and enhance the effectiveness of chemotherapy and radiotherapy, which tend to be less effective in individuals who continue smoking.^41^^,^^42^ As a future perspective, lung cancer teams could be trained in smoking cessation methods to support inpatient care and provide follow-up after discharge. Smoking behavior and treatment data could be integrated into electronic medical records, allowing easy access to relevant clinical variables.^43^ Our results also highlight the necessity of integrating clinical information when receiving PWS diagnosed with lung cancer in SCS, to better understand and support women and men with active tobacco use who intend to quit after diagnosis. Despite the study limitations, our findings have important implications for smoking cessation efforts, particularly in the context of France’s future lung cancer screening pilot program, in which integrating targeted cessation support could enhance patient outcomes.

In conclusion, our study indicates a need for further research into targeted interventions by sex to evaluate the efficacy of smoking cessation strategies in patients with lung cancer. Tailored interventions that integrate mental health support, social reinforcement strategies, and personalized pharmacotherapy might improve cessation outcomes. Future efforts could be considered to prioritize incorporating sex-specific smoking cessation support within lung cancer treatment and screening pathways to maximize both quitting success and overall health outcomes.

## CRediT Authorship Contribution Statement

**Anne-Laurence Le Faou:** Conceptualization; Methodology; Software; Validation; Formal analysis; Investigation; Resources; Data curation; Writing - original draft; Writing - review & editing; Visualization; Supervision; Project administration; Funding acquisition.

**Dalia Alleaume:** Software; Validation; Formal analysis; Writing - original draft; Writing - review & editing; Visualization.

**Ingrid Allagbé:** Conceptualization; Methodology; Software; Validation; Formal analysis; Writing - original draft; Writing - review & editing; Visualization.

## Disclosure

The authors declare no conflict of interest.
